# Influence of Printing Orientation on the Flexural Strength of Different Light-Cured Resins Manufactured with Two 3D Printers: In Vitro Study

**DOI:** 10.3390/ma18133029

**Published:** 2025-06-26

**Authors:** Maria Francesca Sfondrini, Federica Gariboldi, Michael Cerri, Claudia Todaro, Maurizio Pascadopoli, Giulia Casiraghi, Andrea Scribante

**Affiliations:** 1 Unit of Orthodontics and Pediatric Dentistry, Section of Dentistry, Department of Clinical, Surgical, Diagnostic and Pediatric Sciences, University of Pavia, 27100 Pavia, Italy; 2 Private Practice, 29011 Borgonovo Val Tidone, Italy; 3 Unit of Dental Hygiene, Section of Dentistry, Department of Clinical, Surgical, Diagnostic and Pediatric Sciences, University of Pavia, 27100 Pavia, Italy

**Keywords:** 3D printing, resin, supports, printer, software, orientation, flexural strength

## Abstract

Three-dimensional printing is rapidly growing in applied dentistry. In order to print faster, increase workflow, and minimize the consumption of resin material, it is important to use the right printer and the correct printing orientation. The objective of the present report is to analyze the flexural strength of specimens realized with two different dental light-curing resins (Keyguide and C&B) obtained from two different Digital Light Processing (DLP) 3D printers. Different printing orientations (0°, 45°, and 90°) were evaluated. 3D Builder, MeshMixer, RayWare, and Chitubox software were used to design the resin specimens. A total of 15 Keyguide and 15 C&B specimens in the shape of a rectangular parallelepiped, with dimensions of 2 mm × 2 mm × 25 mm, were obtained with the Sprintray Moonray S 3D printer, and the 15 Keyguide and 15 C&B specimens presented the same characteristics as those printed using the Moon Night printer. Prior to sample printing, a calibration protocol (tolerance test and dimensional accuracy test) was performed using RayWare software. This procedure allowed compensation for resin shrinkage or expansion, thus ensuring dimensional consistency in all printed samples. Each resin specimen, after printing and post-processing (MoonWash 2 and MoonLight 2), was subjected to a mechanical test with a universal testing machine. After breaking the specimen, the flexural strength values were recorded with computer software (Bluehill, Instron Corporation, Canton, MA, USA). According to the results obtained, the printing orientation of the specimens does not affect the flexural strength of the two materials examined. However, at the maximum load, some differences emerged for both materials printed with the Moon Night printer, depending on their build angle. Both light-cured resins tested had a higher maximum load resistance when printed with the newer Moon Night printer. This result could be due to the Moon Night printer’s better construction characteristics compared to those of the Sprintray or to issues related to the dimensional calibration of the specimens.

## 1. Introduction

Three-dimensional (3D) printing, also known by the term additive manufacturing (AM), rapid prototyping [1], layered manufacturing [2], or slicing [3], is a rapidly developing technology that has gained wide acceptance and application in the medical field [4], particularly in dentistry [5], as it allows objects and models to be created in a short time, simultaneously and without the need for dental labor, starting from three-dimensional digital images. In the past, the main limitation to the use of 3D printers by a private dentist was the cost, but now, these devices are no longer limited to industrial use; it is an equipment that can be used directly at the dental office [6] since it is a simple machine and compact in size, with an acceptable cost for professional dentists [7,8]. Three-dimensional printers are integrated into dental training to make models for teaching dental anatomy and for practicing procedures such as fillings and root canal treatments, replacing the use of extracted teeth that are prone to contamination [9,10]. In endodontics, 3D-printed guides based on CBCT data are used to find highly calcified canals in non-surgical root canal treatment and for an apicoectomy of posterior teeth in surgical endodontics [11]. Metal structures that can be 3D-printed include removable partial dentures, overdentures, fixed prosthetic structures, and metal implants [12]. Three-dimensional printing of dental crowns and bridges is one of the most appealing applications of 3D printing technology in dentistry [13]. In oral surgery, CBCT, in combination with 3D printing, can also be used to create drilling or cutting guides that make surgery more predictable and quicker, as well as reducing its invasiveness [14]. CAD/CAM osteotomy and repositioning guides are used to achieve virtual 3D planning of the patient’s case for operational simplification and reduction in surgical errors. Biomedical printing and direct metal laser sintering technologies can lead to prefabricated titanium miniplates to be used for screw fixation after bone repositioning [15]. In orthodontics, the printer is often used for the production of surgical guides for the safe, studied, and controlled insertion of miniscrews, even in difficult locations, to save noble, nervous, and vascular structures [16]. Another use is for orthodontic templates to carry out indirect orthodontic bonding, increasingly adopted to shorten the time spent in the chair [17]. Patients’ growing desire for “invisible” alternative treatments has contributed to the spread of mass-produced clear aligners that are manufactured based on traditional impressions or digital scans of the patient’s teeth. With the advent of ever faster and better-performing 3D printers, it will be possible to switch from an STL file to a 3D-printed aligner directly in a few hours under the clinician’s guidance, shortening production times and costs [18]; gnathological bites [19,20] and vacuum formed retainers [21] can be printed using this technique, along with simple data processing software, resulting in very low production times and costs for both the patient and the operator.

The most common 3D printing techniques can be summarized into three types: stereolithography (SLA), Digital Light Processing (DLP), Polyjet, which includes the powder binder printer (PBP), PhotoPolymer Jetting (PPJ), Fused Deposition Modeling (FDM), Selective Laser Melting (SLM), and Selective Laser Sintering (SLS) [22]. This study focused exclusively on DLP 3D printing technology due to its widespread use in dental applications, particularly for producing high-resolution and biocompatible resin components. DLP systems offer faster build times and improved detail reproduction compared to other photopolymerization techniques, making them clinically relevant. SLA and FDM differ significantly in their printing mechanisms, resolutions, and material properties: SLA achieves smoother surfaces but slower print times, while FDM uses thermoplastic filaments, which are less suitable for fine dental structures. These differences in material composition, layer bonding, and polymerization processes could affect mechanical properties such as flexural strength [23].

There are many studies in the literature that have evaluated the deflection, maximum load, flexural strength, and elastic modulus of dental materials; however, these studies have always investigated the behavior of restorative materials traditionally used in dentistry, such as different generations and components of composites. Other studies have compared the flexural strength and Weibull characteristics of milled zirconia produced by additive stereolithography techniques [24]. In a study, Keerthana B et al. evaluated the flexural strength of two different glass ionomer cements after immersion in juice [25]. To date, few studies have investigated the topic regarding the different mechanical properties of 3D-printed specimens in dentistry. In 2020, a research was conducted to evaluate and compare the mechanical properties (flexural strength and surface hardness) of different materials and technologies for the fabrication of prostheses obtained by three different methods: polymerization, Computer-Aided Designing/Computer-Aided Manufacturing (CAD/CAM) technology, and 3D printing; it was seen that CAD/CAM materials had better mechanical properties than heat-cured and 3D-printed materials [26]. In 2022, a systematic literature review and meta-analysis compared the physical and mechanical properties of 3D-printed temporary crowns and FDP resin materials with CAD/CAM-milled materials and conventional temporary resins and found that 3D-printed temporary crown and FDP resin materials have superior mechanical properties but inferior physical properties compared to CAD/CAM milling and other conventionally fabricated materials; 3D-printed temporary crowns and FDP materials can be used as an alternative to conventional and CAD/CAM-milled temporary materials in the long term [27]. In January 2023, a study was conducted with the purpose of comparing the flexural strength of CAD-CAM-milled, 3D-printed, and conventional compression-molded base resin (DBR). CAD-CAM-milled DBRs showed the highest flexural strength compared to conventional compression-molded or 3D-printed DBRs [28].

There are various factors that influence molded artifacts, thus affecting accuracy, processing time, and material properties such as the ultimate tensile stress, modulus elasticity, yield strength, impact strength, and fatigue-induced residual [29]. For SLA or DLP manufacturing processes, the build angle indicates the direction in which the object is cut during the build-up process [30]. The optimal build angle should provide a self-supporting geometry and thus require minimal support structure during the printing process [29].

Previous studies have demonstrated that build orientation in DLP 3D printing can significantly influence the flexural strength of dental resins, with variations observed across different materials and orientations. These findings underscore the importance of systematically evaluating the mechanical performance of 3D-printed dental resins concerning build orientation to inform clinical applications and material selection. On the other hand, previous studies evaluating the effect of build angle on dimensional accuracy in DLP printing have reported minimal or inconsistent impacts, especially when proper calibration protocols are followed. It was hypothesized that orientation might also have a limited effect on flexural strength. However, mechanical behavior involves interlayer bonding and polymerization dynamics, which are not fully captured by dimensional accuracy alone. Therefore, this study aimed to test whether variations in build angle significantly affect flexural performance [31,32,33]. In the literature, there are still no conclusive results for the optimal build angles in different dental applications. A study by Quintana et al. showed that tensile stress and modulus of elasticity are not significantly influenced by axis and position, but layout settings have a significant effect on both properties. Samples built on a corner showed better performance than the other layout [34]. Another study showed that the direction of the molded layer perpendicular to the direction of loading is better than a parallel direction in terms of the compressive strength of the material [35]. A study by Alharbi evaluated the influence of build angle and substrate configuration on the dimensional accuracy of full-coverage dental restorations molded using SLA technology, and the results of the study revealed that both factors influence the size and accuracy of molded parts [36]. In their study, Unkovskiy et al. evaluated the influence of printing parameters on the flexural properties and precision of material samples, prismatic in shape and of the same size as those in the present study, which were printed with an SLA 3D printer. The print orientation influences the printing accuracy [37].

Once the printing phase is completed, the post-processing steps can improve the performance of printed samples at an increased cost and greater time consumption. UV and/or microwave post-curing can improve the modulus of elasticity and ultimate strength of the samples, while increased laser power can also increase the strength of the sample.

However, while several studies have investigated the dimensional accuracy or biocompatibility of dental resins, few have focused on their mechanical behavior—especially flexural strength—under different printing orientations. This is particularly relevant in DLP 3D printing, where the build angle can influence polymerization and interlayer bonding. Therefore, this study aims to address this gap by analyzing how the print orientation affects the flexural performance of two commonly used dental resins. The purpose of this study is to analyze the flexural strength of a series of 2 mm × 2 mm × 25 mm light-cured resin samples based on two different materials obtained from two different DLP 3D printers and to evaluate whether the flexural strength of the products can be affected by the different printing orientations (0°, 45°, and 90°) and the use of a different printer. The null hypothesis of this study is that there are no significant differences among the flexural strengths of the various groups tested.

## 2. Materials and Methods

### 2.1. Sample Preparation

The materials and tools used in this study are summarized in the following table (Table 1):

The two resin materials chosen and used for printing the specimens in this phase of the experiment were Keyguide (Keystone Industries GmbH Stockholzstr., Singen, Germany) and C&B (NextDent, Centurionbaan 190, Soesterberg, The Netherlands). Keyguide is a rigid, biocompatible photopolymerizable 3D printing resin (Class I), used in DLP 3D printers using wavelengths between 385 nm and 405 nm. This resin is composed primarily of urethane acrylate oligomers, with additional dimethacrylate monomers such as HEMA and hexanediol diacrylate. It also contains photoinitiators (TPOs) and stabilizers (e.g., MEHQ). According to the manufacturer’s technical data, its flexural strength reaches approximately 106–140 MPa, with a flexural modulus of around 2400–2500 MPa and a Shore D hardness of 95. These characteristics reflect a rigid and biocompatible resin, suitable for intraoral surgical guides. It has to be used with nitrile or latex gloves [38].

The C&B resin used in this study is a light-curable photopolymer for the fabrication of temporary crowns and bridges using 3D printing technologies; it is a material composed of a methacrylic oligomer, glycol methacrylate, and phosphine oxide. It is a high-molecular-weight acrylic resin that has the following advantages: ease of use, versatility, high aesthetic results, even at a reduced thickness, resistance to abrasion and fracture, low bacterial plaque adhesion, and high biocompatibility. High strength gives Crown & Bridge resin greater strength and stability during construction, resulting in less shrinkage and curling than similar products [39]. According to the manufacturer’s technical data, the material exhibits a flexural strength of 107 MPa. It also shows low water sorption (54 µg/mm^3^) and solubility (5.9 µg/mm^3^).

**Figure 1:**
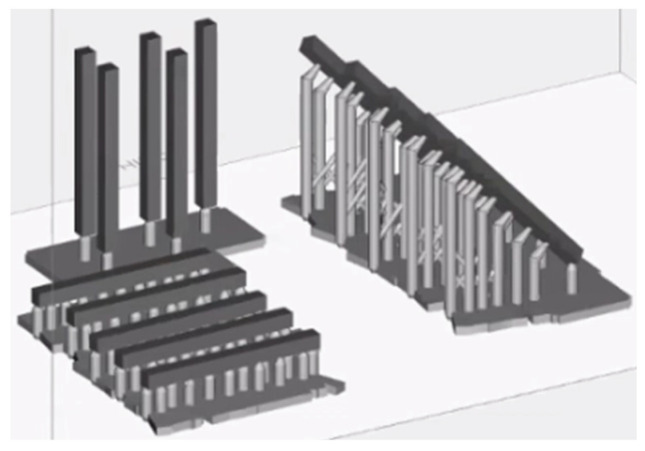
A rectangular parallelepiped .stl file imported into RayWare 2.8.9 software to finalize and add print media; print media were added to all specimens at different angles (0°, 45°, and 90°).

A 2D square was selected with 3D Builder software version 20.0.4.0. (Microsoft, Redmond, WA, USA) and transformed into a 3D cube, which, in turn, was modified with MeshMixer (Autodesk, Inc., San Rafael, CA, USA) software to make it a rectangular parallelepiped with the dimensions of 2 mm × 2 mm × 25 mm. The software was used to create the .stl files of the 15 Keyguide material specimens and the 15 C&B material specimens. Finally, RayWare 2.8.9 software (SprintRay, Inc., 2710 Media Center Drive, Los Angeles, CA, USA) was used to finalize and add the print media to the Keyguide and C&B specimens and then print them using the Sprintray Moonray S Vertysystem 3D printer (SprintRay, Inc., 2710 Media Center Drive, Los Angeles, CA, USA) (Figure 1).

The sample size was calculated using the following equation:

n=2Zα2+Zβ 2σ2∆2

where Z_α/2_ = 1.96 for a confidence interval of 5% (α = 0.05), Z_β_ = 0.84 for a power of 80% (1 − β = 0.80), σ = 14.1 MPa (standard deviation), and Δ = 25 MPa (expected difference), considering the results from previous studies [26]. Five specimens per group were required according to the calculation. The 5 samples for each type of print were created and entered into the reference plane based on the angle. A priori sample size calculation was performed based on preliminary data and anticipated effect sizes. This analysis determined that 5 specimens per group would be sufficient to achieve adequate statistical power for detecting meaningful differences in flexural strength. Such software also allows a manual or automatic choice of media, as well as their density and size. In the current study, the automatic print media insertion parameter was chosen; additional media were added manually in cases where it was necessary to achieve the most accurate printing possible. The supports allow the printing process to be guaranteed for artifacts that have negative or positive angles but less than or equal to 45°, and if 20 μm quality printing is selected, they also facilitate the detachment of the design from the aluminum plane. RayWare uses an intelligent media generation algorithm, inserting a minimum number of media. Flat models do not require supports, but in the present study, they were still inserted to homogenize the samples. In the above study, 4 supports were placed in the 45° samples, along with 1 support in the 90° sample and 4 supports in the 0° samples.

**Figure 2:**
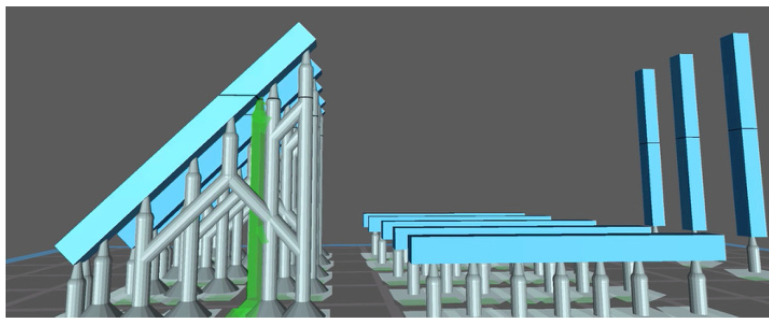
Chitubox screenshot showing the automatic addition of supports; it is possible to choose to add more media manually by pre-screening their future allocation in green.

At this point, the printer, Sprintray Moonray S, and the chosen material (Keyguide or C&B) were selected, and the printing resolution and layer thickness (expressed in μm), namely, the increments along the *Z*-axis, were chosen; a 50 μm resolution was used [37]. As for the Sprintray Moonray S 3D printer, the tank consists of a machined aluminum plate with a vacuum cover. The printer is pre-calibrated and must be used at 18°–27°. Prior to printing the part, to account for potential differences between the RayWare and Chitubox slicing environments, dimensional calibration tests (Tolerance and Dimensional Accuracy) were performed independently for each printer/software combination. Based on the outcomes, adjustments were made within each slicing software to ensure dimensional consistency and reduce variability between the printed specimens, despite using different platforms. Once these preliminary tests were completed, the design was printed, the printer plate and resin reservoir were removed, and the molded object was removed from the platform using a scraper with a blade. With regard to the design created with MeshMixer and imported into the Chitubox software (CTB Systems, Zhongcheng Future Industrial Park, Shenzhen, China), the five specimens for each type of print were created and then inserted into the reference plan. The substrates were inserted automatically, and additional substrates were added manually where necessary to achieve the most accurate print possible (Figure 2). This is because the resin layers between one substrate and the next are not supported by anything; so, in order to achieve greater stability and avoid possible collapse, it is necessary to add an additional intermediate substrate between the two that are already present. The supports allow the printing process to be guaranteed for artifacts with negative or positive angles less than or equal to 45° and also facilitate the detachment of the project from the aluminum plate. Chitubox uses an intelligent algorithm for generating supports, inserting a minimum number of them. Flat models do not require supports, but in the present study, they were nevertheless inserted for sample homogeneity.

### 2.2. Three-Dimensional Printing

The printing of the specimens inside the 3D printer is performed upside down. The easiest specimens to print are those that are at a 90° angle, as they require a single substrate; the layers are layered without difficulty, and there are no dimensional changes or sags [37,40]. The 45° specimens can be molded without any particular dimensional changes as the resin is added layer by layer with incremental additions, allowing the resin specimen to grow and develop without printing errors [37,40]. The most complex specimens to print are those at 0° since between one support and the next, the layered resin has no support, and growing in height could lead to a locus minoris resistentiae, thus causing the resin to collapse; adding extra supports gives greater stability but modifies the dimensional and thickness parameters of the specimen. In the above study, supports were placed as follows: 4 in the design with a 45° inclination, 1 in the design with a 90° inclination, and 4 in the design with a 0° inclination. The height of the layers was set to 0.050 mm. The chosen material was then loaded into the tank of the Moon Night 3D printer (Vertysystem, Altavilla Vicentina (VI)—Italy). The printer, material profile, and layer thickness were selected. Six initial layers were printed, which were attached to the printing plate and used to create the pedestal of the print supports. The ‘cure time’ was set to 1,600 s, while the ‘base cure time’ was set to 35,000 s; the latter exposure time ensured that the initial layers remained attached to the printing plate without dropping the printed specimen. These settings followed the resin manufacturer’s validated recommendations and were not altered. The choice of a 0.050 mm layer height and the exposure parameters were based on the resolution, dimensional fidelity, and print reliability. Preliminary test prints and calibration procedures were performed to confirm that the chosen parameters resulted in accurate and stable prints without overcuring or delamination. Having printed the initial 6 layers, the printer then started printing 1 layer every 5 μm. When everything was ready to print, the computer with the Chitubox software communicated 600 images with the 3D printer, and on the LCD screen, 600 images of this type were reproduced (white dots on a black background, indicating the rising resin layers), and each image was reproduced for a certain number of seconds, representing the curing time required for the chosen resin. This previsualization serves to detect any errors: if white dots instead of white stripes appear next to each other from one layer to the next, then note that the print may fail at these points. This is because the addition of resin must always be progressive; new white dots or even stripes must never appear from nowhere. Once the printing stage is complete, the printer platen and resin reservoir are removed. It is necessary to detach the printed object from the platform using a metal-bladed scraper to lift the edges of the supports and then detach the entire artifact.

### 2.3. Post-Processing

Post-printing processes, such as washing and photopolymerization of 3D resins, are essential steps to ensure a perfect final artifact. In the above study, MoonWash 2 (Vertysystem, Altavilla Vicentina (VI)—Italy), a 3D printing resin cleaner, was used in the post-production phase. It cleans the uncured surface resin from the 3D printer using special chemicals, including isopropyl alcohol (IPA). The manufacturer recommends using three different types of substances in combination with MoonWash 2: Vertys Splash, Vertys Spray Dry, and Vertys Flusher (Vertysystem, Altavilla Vicentina (VI)—Italy). All of these products are used with the cleaning kit supplied by the 3D printer company. MoonLight 2 (Vertysystem, Altavilla Vicentina (VI)—Italy) is an additional post-production machine that enables UV photopolymerization of the 3D-printed artifact to achieve curing.

At the end of these processes, the substrates can be removed with the use of cutters.

This procedure was repeated with both printers for the 5 samples with a print orientation of 0° (horizontal pull direction) and then replicated for the other 5 with an orientation of 45° and the remaining ones with a 90° orientation (vertical pull direction) for both the Keyguide and C&B materials. These orientations were chosen following Unkovskiy’s selection criteria [37]. These three angles were selected because they represent standard reference orientations in the additive manufacturing literature, corresponding to horizontal (0°), diagonal (45°), and vertical (90°) layer stacking. This range allows for a meaningful comparison of the impact of build direction on mechanical performance while maintaining clinical relevance and methodological simplicity. At the end of this first phase of artifact creation, 15 specimens of Keyguide material and 15 specimens of C&B material in the shape of a rectangular parallelepiped measuring 2 mm × 2 mm × 25 mm in height, width, and length, respectively, were obtained by means of the Sprintray Moonray S 3D printer, and 15 specimens of Keyguide material and 15 specimens of C&B material with the same characteristics were obtained by means of the Moon Night printer.

### 2.4. Mechanical Testing

**Figure 3:**
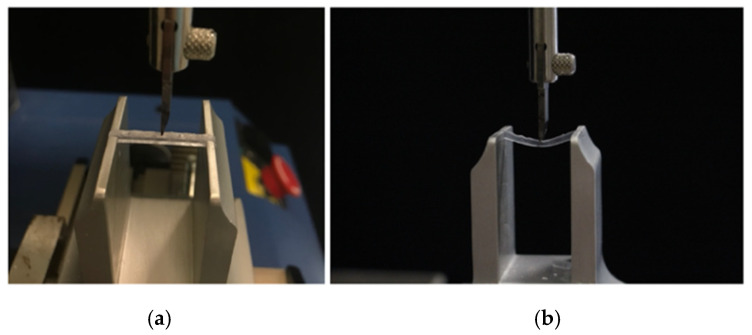
Details of the specimen housing in the Instron: specimen just before the start of application of the compressive load (**a**) and specimen in bending during the mechanical test (**b**).

The arteficts were divided into 3 different categories, each consisting of 5 prisms, and then subjected to mechanical testing. Each resin material specimen, after printing and post-print processing steps, was subjected to a mechanical test with a universal testing machine: the rectangular parallelepiped specimen was placed in an appropriate aluminum holder (Figure 3).

**Figure 4:**
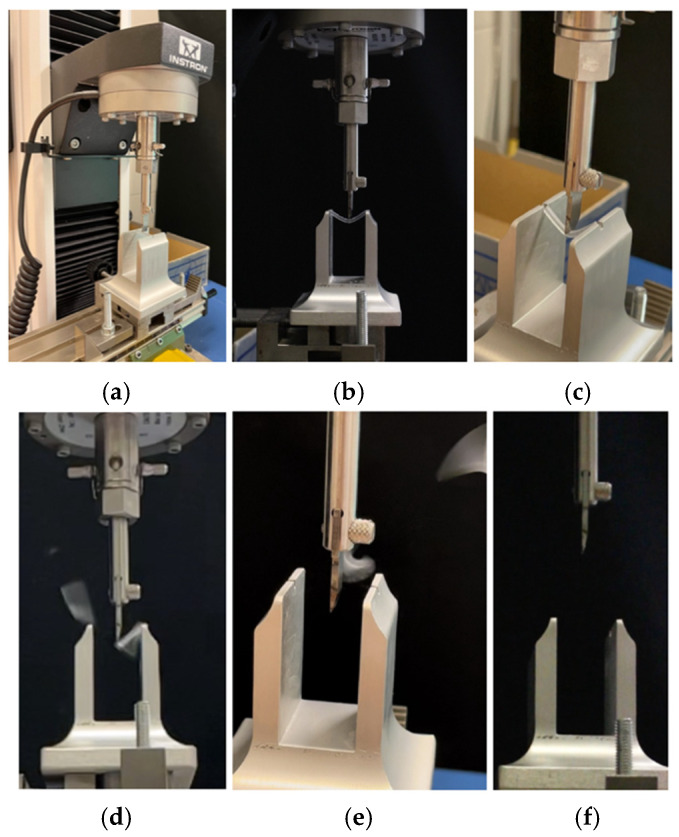
Performing the mechanical test: specimen housed in the support (**a**); start of the mechanical test and specimen in bending (**b**); elastic behavior of the resin specimen subjected to the mechanical test (**c**); exceeding the elastic threshold and breaking the specimen into several pieces (**d**,**e**); and end of the mechanical test (**f**).

**Figure 5:**
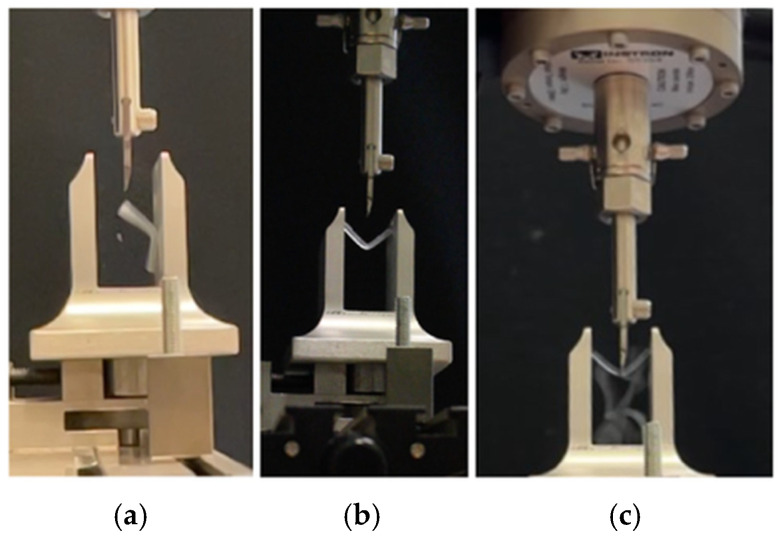
Mechanical test with a specimen that does not break (**a**) and exits the support rails (**b**,**c**). The path of the sample is shown in the case on the right (**c**).

A universal testing machine (model 3343, Instron Corporation, Canton, MA, USA) was used in order to apply a compressive load to resin specimens. Its single-column system is ideal for tensile and/or compressive applications where tests use less than 5 kN. Regarding the features of the universal machine, the force range is 1 to 200. The data acquisition frequency is 500 Hz, and the machine is compatible with Bluehill Software 2. There is automatic transducer recognition for load cells and strain gauges. The device has a capacity of 1 kN and works at a minimum speed of 0.05 mm/min and a maximum speed of 1000 mm/min; the vertical test space is 1067 mm. The machine has a height of 32.00 cm, a width of 20.00 cm, a length of 18.00 cm, and a weight of 94.00 pounds. The span length between the supports was 21 mm, and the crosshead speed was set to 1 mm per minute. The compressive load was applied with a universal testing machine until the specimen broke or exited the supports (Figure 4 and Figure 5). The test parameters (21 mm support span, 1 mm/min loading rate) were consistent with the general procedure outlined in ISO 178:2019 [41] for three-point flexural testing of rigid plastics. The machine was equipped with a 1 kN load cell. It is acknowledged that the force values recorded at 0.1 mm deflection were, in some cases, below the 5 N threshold, which corresponds to the minimum recommended load (1:200 of the load cell’s range) for optimal accuracy. This is considered a methodological limitation of the present study.

After breaking the specimen, the flexural strength values were recorded with computer software (Bluehill, Instron Corporation, Canton, MA, USA). After collecting the data, the flexural strength (*σ*) and elastic modulus (E) were calculated. The flexural strength (σ, in MPa) was calculated from the maximum load (F) using the following formula:

$$\sigma =\frac{3FL}{2bd^{2}}$$

where L = 21 mm is the support span and b = d = 2 mm are the cross-sectional dimensions of the samples. This is simplified as follows:

$$\sigma =\frac{63F}{16}$$

### 2.5. Statistical Analysis

Data analysis was carried out with R software (R version 3.1.3, R Development Core Team, R Foundation for Statistical Computing, Wien, Austria) by calculating descriptive statistics for each variable that included: mean, standard deviation, median, minimum, and maximum values, measured for each group. The Kolmogorov–Smirnov test was used to assess the normality of distributions. As data were normally distributed, an ANOVA test was then applied, followed by Tukey’s post hoc tests to perform multiple intergroup and intragroup comparisons between the three printing angles (0°, 45°, 90°) on each material, and the performance of the two 3D printers. Linear regressions were performed to evaluate the effect of materials, degrees, and printers on the deflection forces and maximum load. For all tests, significance was set at *p* < 0.05.

## 3. Results

The deflection tests at 0.1 mm and 0.2 mm for this study were chosen for a practical and normative motivation: 0.1 mm and 0.2 mm are small deformations that allow the elastic response of the material to be accurately measured before permanent or plastic deformation occurs. At these levels of deflection, the behavior of the material or system is typically linear elastic, thus being easily modeled and compared [42]. Both flexural strength and elastic modulus were evaluated to comprehensively assess the mechanical performance of the tested resins. Flexural strength reflects the material’s maximum resistance before failure under bending stress. The elastic modulus indicates the material’s stiffness or its ability to resist elastic deformation under load [43].

### 3.1. Deflection at 0.1 mm

**Figure 6:**
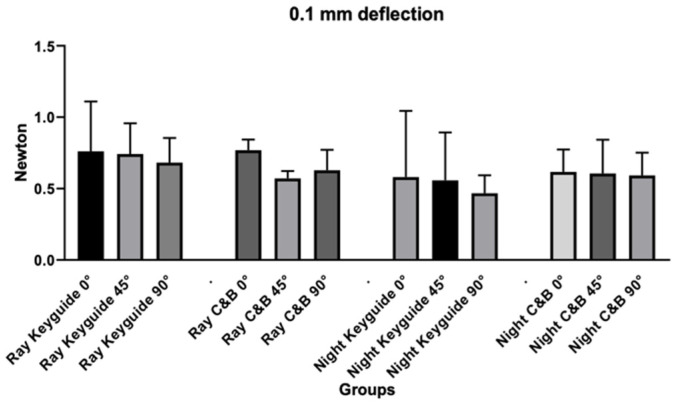
Graphic obtained from the analysis of variance for the deflection at 0.1 mm.

Regarding 0.1 mm deflection values (Table 2 and Figure 6), the ANOVA test revealed no significant differences for the variables tested in relation to the deflection at 0.1 mm.

### 3.2. Deflection at 0.2 mm

**Figure 7:**
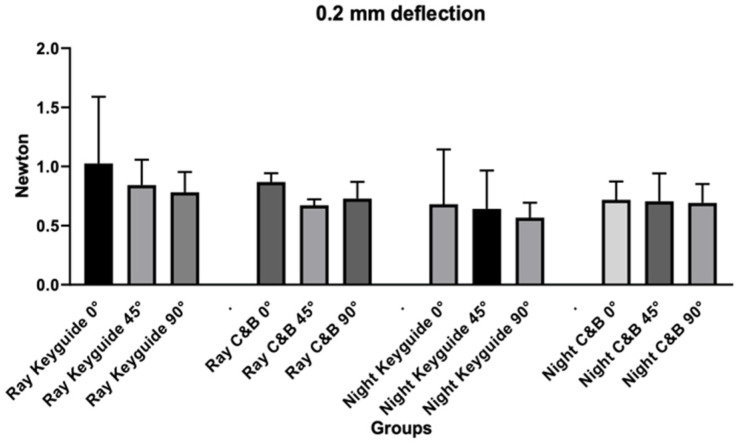
Graphic obtained from the analysis of variance for the deflection at 0.2 mm.

Regarding 0.2 mm deflection values (Table 3 and Figure 7), the ANOVA test revealed no significant differences for the variables tested in relation to the deflection at 0.2 mm.

### 3.3. Maximum Load

**Figure 8:**
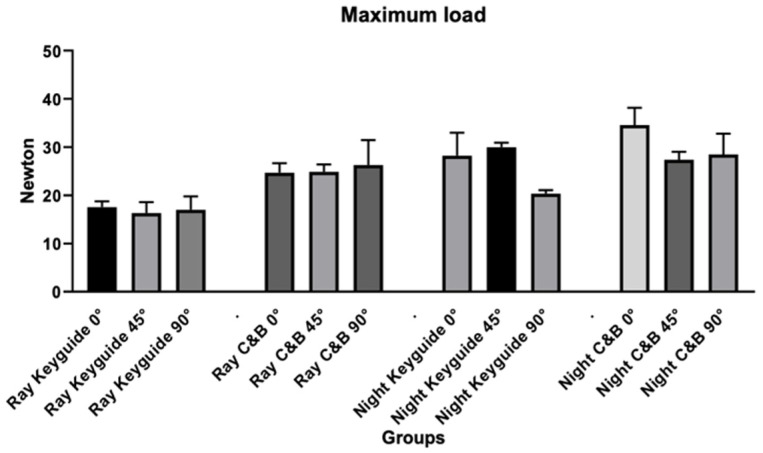
Graphic obtained from the analysis of variance for the maximum load.

Regarding maximum load values (Table 4 and Figure 8), the ANOVA test revealed significant differences for the variables tested in relation to the maximum load. Tukey’s post hoc test revealed significant differences for the variables tested in relation to the maximum load.

**Figure 9:**
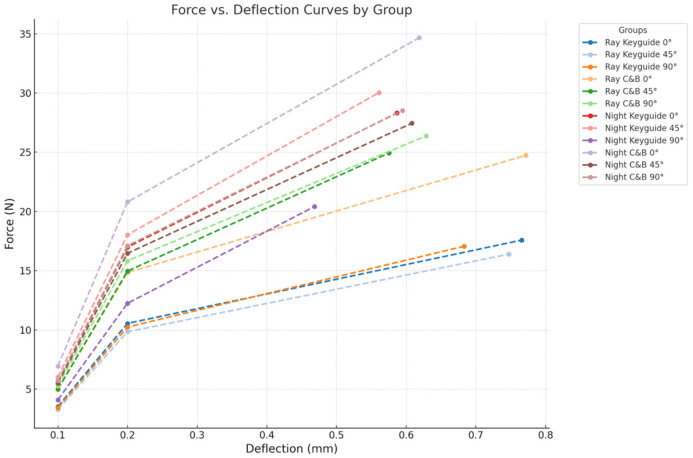
Force versus deflection curves for all groups, estimated at 0.1 mm and 0.2 mm of deflection and maximum load.

In addition to the statistical evaluation of maximum load values, Figure 9 presents the force vs. deflection curves for all experimental groups. These curves were constructed using estimated forces at 0.1 mm and 0.2 mm deflection, along with the recorded maximum load. The graphical representation highlights differences in mechanical response among materials, printing angles, and printer types.

### 3.4. Flexural Strength

In addition to the maximum load values (N), the corresponding flexural stress values (MPa) were calculated and are shown in Table 5. These allow for a standardized comparison with other studies and manufacturer-reported material properties. Regarding flexural strength values, the ANOVA test revealed no significant differences for the variables tested.

### 3.5. Modulus of Elasticity (E)

The modulus of elasticity values (MPa) were calculated and are shown in Table 6. These allow for a standardized comparison with other studies and manufacturer-reported material properties. Regarding the modulus of elasticity values, the ANOVA test revealed no significant differences for the variables tested.

**Figure 10:**
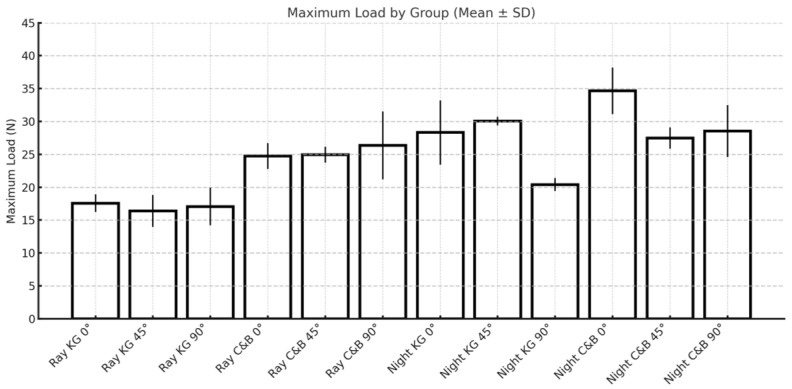
Influence of printing orientation on the maximum load.

Pooling the behavior of the two materials printed at the three different angles, it can be seen that there is actually no relevant influence of the printing orientation on the maximum load (Figure 10).

**Figure 11:**
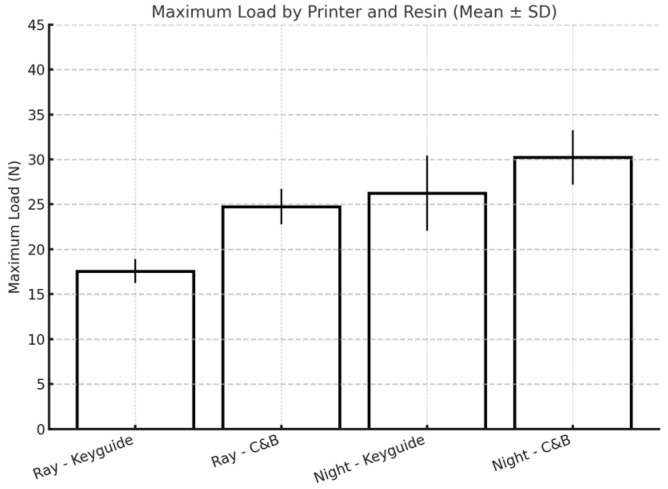
Influence of material type on the maximum load.

The type of material has no effect on the deflection at 0.1 mm and 0.2 mm, but it does affect the maximum load values. KeyGuide and C&B materials resist maximum load differently; in particular, C&B seems to have slightly higher deflection values than KeyGuide (Figure 11) with both printers.

**Figure 12:**
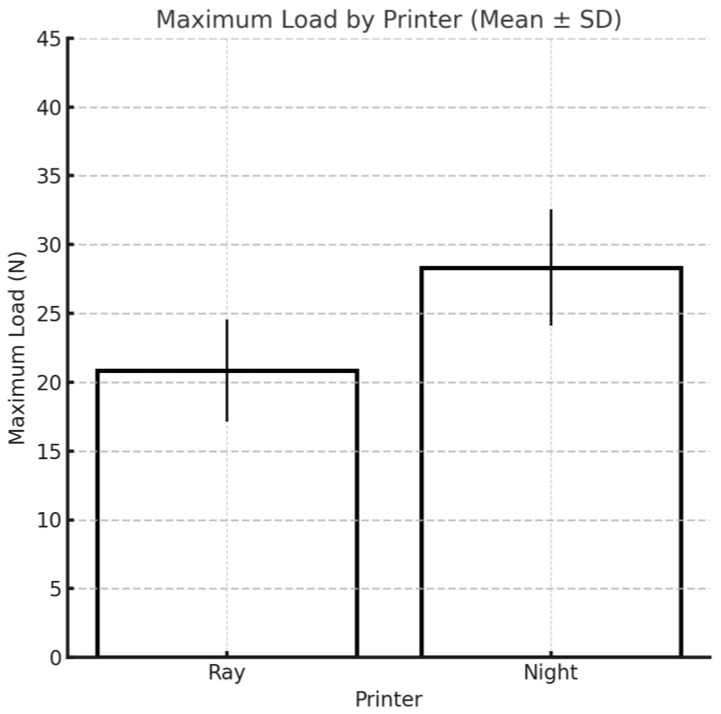
Influence of printer type on the maximum load.

Overall, the maximum load resistance of both materials tested is significantly higher when the materials are printed with the Moon Night printer, which is the most recently manufactured printer (Figure 12).

### 3.6. Linear Regressions

From the linear regressions (Table 7), it can be deduced that with regard to the deflection at 0.1 mm:

- For the same printer used and printing angle chosen, there is no significant influence of the material on the deflection at 0.1 mm (*p* > 0.05);
- For the same printer used and material chosen, there is no significant influence of the printing angle on the deflection at 0.1 mm (*p* > 0.05);
- For the same material used and printing angle chosen, there is a significant influence of the printer used on the deflection at 0.1 mm (*p* < 0.05).

It can be deduced from the linear regressions (Table 7) that with regard to the deflection at 0.2 mm:

- For the same printer used and printing angle chosen, there is no significant influence of the material on the deflection at 0.2 mm (*p* > 0.05).
- For the same printer used and material chosen, there is no significant influence of the printing angle on the deflection at 0.2 mm (*p* > 0.05).
- With the same material used and printing angle chosen, there is a significant influence of the printer used on the deflection at 0.2 mm (*p* < 0.05).

It can be inferred from the linear regressions (Table 7) that with regard to maximum load:

- For the same printer used and printing angle chosen, there is a significant influence of the material on the maximum load (*p* < 0.05);
- With the same printer used and material chosen, there is no significant influence of the printing angle on the maximum load (*p* > 0.05);
- With the same material used and printing angle chosen, there is a significant influence of the printer used on the maximum load (*p* < 0.05).

## 4. Discussion

Three-dimensional (3D) printing technology produces three-dimensional objects based on patterns previously designed on a computer. This new production model is consistent with the assumptions of Industry 5.0, based on automation and digitization, which perfectly correlates with the assumptions of 3D printing technology [44]. An important element of Industry 5.0 is 3D printing technology because of its favorable environmental orientation and production flexibility. Three-dimensional printing technology uses recycled materials such as powders. Therefore, it can be part of a circular economy, contributing to environmental protection.

This in vitro study focused on the innovative topic of analyzing the different mechanical properties of resin materials printed with DLP 3D printing technology, with a special focus on the parameter of flexural strength. Flexural testing was chosen because it simulates the combined tensile and compressive stresses experienced by dental restorations during clinical function. This type of loading is particularly relevant for temporary crowns, bridges, and surgical guides, which are subject to masticatory forces and off-axis loading in the oral environment. Compared to isolated tensile or shear tests, flexural tests provide a more realistic assessment of a material’s ability to withstand functional intraoral stresses [42]. This study was carried out by printing specimens using two different 3D printers (Sprintray Moonray S and Moon Night), with two different materials each (Keyguide and C&B), at three different angles (0°, 45°, and 90°). In light of the linear regressions obtained, it would appear that the printing orientation of the specimens does not affect the flexural strength of the two materials examined in any way. However, from Tukey’s post hoc test, at the maximum load, some differences emerged for both materials printed with the Moon Night printer depending on their build angle, specifically, between Keyguide at 0° and 90°, between C&B at 0° and 45°, and between Keyguide at 45° and 90°. Although significant differences in the maximum load were observed among Moon Night-printed specimens with different build orientations, these differences did not consistently translate into significant variations in flexural stress (MPa), which accounts for the cross-sectional dimensions. Notably, the significantly lower maximum load observed in Moon Night-printed Keyguide specimens at 90° may be attributed to the layer orientation being perpendicular to the direction of the applied load. This configuration can reduce interlayer cohesion and create weak planes that are more susceptible to delamination under bending stress. This observation is consistent with previous findings that vertically printed specimens often exhibit lower mechanical resistance due to suboptimal layer-to-layer bonding. Therefore, while the printing orientation appears to influence the absolute breaking force in some conditions, its effect on normalized mechanical performance remains limited within the scope of this study. This finding may support greater clinical flexibility in selecting build orientations based on practical factors—such as model geometry, print efficiency, or post-processing ease—without compromising mechanical reliability. This improved mechanical performance suggests that Moon Night-printed resins may be particularly well-suited for clinical applications requiring enhanced strength and dimensional stability, such as temporary crowns, bridges, and surgical guides that must withstand repetitive occlusal loading and insertion forces. In terms of flexural strength, the C&B resin showed consistently higher mean values compared to Keyguide, with the highest values recorded for specimens printed with the Moon Night printer at a 0° orientation (136.5 MPa). For the elastic modulus, the values also varied between resins and printers, with the highest value observed for C&B at 45° printed with the Moon Night printer (2353.3 MPa). These findings establish the mechanical trends among groups prior to inferential statistical evaluation. The most interesting results of this study concern the influence of printer type on all the variables tested: from the linear regressions, it would appear that printer type has an influence on the deflection at 0.1 mm and 0.2 mm; however, no statistically significant results emerged from Tukey’s post hoc test. Based on the results, it would appear that the type of printer has a statistically significant effect on the maximum load. The finding of such a result could depend on the better-performing construction characteristics of the Moon Night printer compared to the Sprintray printer. The Moon Night printer may incorporate improved light distribution systems, higher-resolution LCD components, or more efficient software calibration algorithms.

Recent studies have confirmed that the mechanical performance of 3D-printed orthodontic aligners is significantly influenced by both the printing technology and post-processing protocols employed. In particular, variables such as printing orientation (0°, 45°, and 90°) and resin thickness have been shown to affect flexural strength. A 2025 study by Khalil et al. demonstrated that although no statistically significant differences were found among the various printing orientations, aligners printed with a greater thickness (0.7 mm) exhibited significantly higher flexural strength compared to those with a thickness of 0.5 mm [45]. Additional research has highlighted the role of post-curing conditions (particularly curing in an oxygen-free environment) in enhancing the degree of conversion and the resulting mechanical properties of the material [46]. Neoh et al. reported that centrifugation prior to post-curing in glycerin led to specimens with reduced surface roughness, improved light transmittance, greater hardness, and enhanced color stability. However, this method also resulted in an increase in material thickness, which may necessitate design modifications to ensure proper fit to the dental model [47].

It is necessary to take into account a number of variables not directly related to printer properties that might have influenced the results obtained. When analyzing the differences between the two printers more closely, it would seem that it is the software that matters the most, while the hardware of the two printers is almost identical. There are studies that have evaluated the differences between 3D printers with different hardware. For example, Tsolakis IA et al. compared the accuracy, in terms of truthfulness and precision, of a 3D printer with a liquid crystal display (LCD) versus a Direct Light Processing (DLP) 3D printer for printing dental models. Therefore, two different printers were used in that study in terms of 3D printing technology. It was seen that the DLP 3D printer is more accurate in terms of printing the dental model than the LCD 3D printer; however, both DLP and LCD printers can be used accurately to print dental models for the fabrication of orthodontic appliances [48]. Few studies, however, have investigated the difference in accuracy between two printers with the same hardware but different printing software. The use of two different software programs for sample design and printing may have affected the dimensional calibration of printed specimens, leading to the printing of specimens of different sizes, depending on their degree of expansion or contraction [49,50]. The tolerance and dimensional accuracy tests to be performed with the RayWare software and the printing and tolerance parameters preset with the Chitubox software could have resulted in inaccuracies in the size of the printed specimens. Using one software to design and print the samples with the two different printers could have made the dimensional calibration of the samples more uniform [51]. In summary, the linear regressions performed in this study showed no effect of the printing angle; rather, there was an effect of the printer and an effect of the material only at the maximum load. The observed differences in mechanical performance between Keyguide and C&B resins, as well as between the MoonRay and Moon Night printers, may be partly attributed to variations in resin composition and slicing software calibration. Previous studies have shown that differences in monomer formulation and filler content can significantly influence flexural strength and polymerization behavior [33,52]. Moreover, the use of different slicing software, with their unique exposure settings and layer processing algorithms, can impact dimensional accuracy and interlayer bonding quality. These factors highlight the importance of standardizing not only materials but also software parameters in future investigations.

A limitation of this study involves the use of different slicing and printer software—RayWare and Chitubox—for the two printers. Since each printer is compatible with its own software, it was not possible to isolate the effect of the software from that of the printer itself. Differences in dimensional calibration or print parameters introduced by the software may have influenced the mechanical performance of the printed specimens. Future studies should aim to evaluate the independent impact of slicing software on the quality of the final parts. Printing orientation is another factor that could have affected printing accuracy: for example, the first layers of the 0° specimens require additional exposure time to be light-cured to ensure their secure adhesion to the substrates; the same is true for the 90° specimens, which need to be cured longer to help achieve the predetermined length. Only three printing angles (0°, 45°, and 90°) were tested. Although they represent standard reference orientations, future investigations will aim to assess additional build orientations, such as 30° and 60°, to provide a more detailed understanding of how intermediate angles may influence the mechanical performance of 3D-printed dental resins. Alterations in specimen size could also arise as a result of the removal of support structures, particularly for the 0° and 45° groups, a procedure that inevitably damages specimens of these sizes. Regarding alterations in specimen size, it must be considered that for both printers, the layer height was set to 0.05 mm, which means that variations less than 0.05 mm are not controllable by either printer [53]. All of these variables could have impacted the final measurements of the specimens by causing relatively large deviations that could have resulted in overlapping effects with those of printing. This study focused exclusively on flexural strength and maximum load, which are important indicators of mechanical performance. Other relevant mechanical properties, such as surface hardness, tensile strength, and fatigue resistance (important for dental applications), were not assessed. These aspects should be included in future studies to provide a more comprehensive mechanical characterization of 3D-printed dental materials. A small sample size is commonly used in preliminary in vitro studies, and it may reduce the statistical power and limit the generalizability of the findings, especially when considering the variability of the 3D printing process. Future studies with larger sample sizes, involving other printers and materials, are recommended to confirm and strengthen these observations.

## 5. Conclusions

This in vitro study evaluated the flexural strength of 3D-printed specimens produced using two different resin materials and printed with two printer models and three build orientations. Within the limitations of this study, the results suggest that while printing orientation did not have a consistent effect across all groups, significant differences were observed in specific conditions (particularly with the Moon Night printer), where build angle influenced maximum load values. We found that 90° printing generally exhibited less favorable mechanical performance, particularly with the Keyguide resin, while 0° and 45° orientations often yielded higher flexural strength and better deformation resistance. Flexural strength is a key determinant of the clinical performance of 3D-printed dental devices, particularly those exposed to repetitive mechanical stress, such as temporary crowns, bridges, and aligners. Intraorally, these restorations must endure bending forces resulting from occlusion, parafunctional habits, and the mechanical demands of insertion and removal. Inadequate flexural resistance may lead to deformation, fracture, or premature failure, ultimately compromising fit, function, and patient comfort. As such, evaluating how processing parameters—especially, build orientation—affect flexural behavior is essential to optimize material selection and anticipate clinical longevity.

The C&B resin consistently demonstrated superior mechanical properties compared to the Keyguide resin, regardless of orientation. Furthermore, specimens printed with the Moon Night printer showed higher maximum load and flexural stress values across both materials compared to those printed with the Sprintray MoonRay S. These differences may be attributed to more advanced construction features of the Moon Night printer or a more accurate dimensional calibration during the printing process. Taken together, these findings highlight the importance of selecting the appropriate combination of resin, printer, and printing orientation to ensure the optimal mechanical performance of 3D-printed dental components. Further research should investigate the long-term behavior and clinical relevance of these materials under functional loading conditions. Additionally, future studies should evaluate the mechanical performance of these materials under simulated oral conditions (thermal cycling, humidity, and dynamic loading) to better approximate clinical use. Furthermore, standardizing dimensional calibration through the use of a single slicing software or external calibration references for both printers should be considered in future studies to minimize variability introduced by different software environments.

## Figures and Tables

**Table 1:** Summary of materials and software used in this study.

Product/Software	Manufacturer	Description
**3D Builder 20.0.4.0**	Microsoft, Redmond, WA, USA	Software for transforming 2D images into 3D objects
**MeshMixer 3.5.0**	Autodesk, Inc., San Rafael, CA, USA	3D design manipulation software
**RayWare 2.8.9**	SprintRay Inc., Los Angeles, CA, USA	Software for finalizing specimens printed with the Spintray Moonray S printer and adding print supports
**Chitubox 2.0.8**	CTB Systems, Shenzhen, China	Software for finalizing specimens printed with the Moon Night printer and adding print supports
**Sprintray Moonray S**	SprintRay Inc., Los Angeles, CA, USA	3D Printer
**Moon Night**	Vertysystem, Altavilla Vicentina (VI), Italy	3D Printer
**MoonWash 2**	Vertysystem, Altavilla Vicentina (VI), Italy	Cleaner for post-production
**MoonLight 2**	Vertysystem, Altavilla Vicentina (VI), Italy	UV photopolymerizer for post-production using a universal testing machine
**Instron 3343**	Instron Corporation, Canton, MA, USA	Universal testing machine
**Keyguide**	Keystone Industries GmbH, Singen, Germany	Resin material
**C&B**	NextDent, Soesterberg, The Netherlands	Resin material

**Table 2:** Descriptive statistics of the values of forces measured for a deflection of 0.1 mm. The unit of measurement of the results obtained is in newtons. * indicates that values with the same letters are not significantly different.

Printer	Resin	Printing Angle	Mean	St. Dev.	Min	Mdn	Max	Significance *
**Ray**	**Keyguide**	**0°**	0.76552	0.35668	0.3668	0.92162	1.19133	A
**Ray**	**Keyguide**	**45°**	0.74702	0.21748	0.54416	0.71232	1.01925	A
**Ray**	**Keyguide**	**90°**	0.68293	0.17031	0.51196	0.66893	0.94472	A
**Ray**	**C&B**	**0°**	0.77168	0.0637	0.72083	0.7511	0.84313	A
**Ray**	**C&B**	**45°**	0.57537	0.0488	0.54273	0.55556	0.64761	A
**Ray**	**C&B**	**90°**	0.62863	0.13597	0.47895	0.59233	0.84917	A
**Night**	**Keyguide**	**0°**	0.58666	0.47034	0.04301	0.54978	1.2369	A
**Night**	**Keyguide**	**45°**	0.56091	0.33865	0.1134	0.78646	0.82718	A
**Night**	**Keyguide**	**90°**	0.4682	0.12561	0.31154	0.44316	0.62342	A
**Night**	**C&B**	**0°**	0.6186	0.15179	0.48546	0.56712	0.8767	A
**Night**	**C&B**	**45°**	0.60815	0.24273	0.32892	0.57488	0.98868	A
**Night**	**C&B**	**90°**	0.59475	0.16791	0.35489	0.57898	0.82143	A

**Table 3:** Descriptive statistics of the values of forces measured for a deflection of 0.2 mm. The unit of measurement of the results obtained is in newtons. * means that values with the same letters are not significantly different.

Printer	Resin	Printing Angle	Mean	St. Dev.	Min	Mdn	Max	Significance *
**Ray**	**Keyguide**	**0°**	1.03139	0.57428	0.46581	1.02552	1.85059	A
**Ray**	**Keyguide**	**45°**	0.8475	0.21761	0.64589	0.81193	1.12026	A
**Ray**	**Keyguide**	**90°**	0.78328	0.16969	0.614	0.76826	1.04507	A
**Ray**	**C&B**	**0°**	0.87204	0.06236	0.8227	0.85129	0.94214	A
**Ray**	**C&B**	**45°**	0.67581	0.04845	0.64445	0.6555	0.7478	A
**Ray**	**C&B**	**90°**	0.72885	0.13474	0.58115	0.69302	0.94786	A
**Night**	**Keyguide**	**0°**	0.68691	0.47143	0.1417	0.65075	1.33791	A
**Night**	**Keyguide**	**45°**	0.64573	0.32807	0.21241	0.80845	0.92787	A
**Night**	**Keyguide**	**90°**	0.56829	0.1252	0.41327	0.54335	0.72412	A
**Night**	**C&B**	**0°**	0.71876	0.15131	0.58432	0.66885	0.97555	A
**Night**	**C&B**	**45°**	0.70818	0.24319	0.42961	0.67327	1.0904	A
**Night**	**C&B**	**90°**	0.69504	0.16912	0.4539	0.67918	0.92363	A

**Table 4:** Descriptive statistics inherent to the maximum load. The unit of measurement of the results obtained is in newtons. * means that values with the same letters are not significantly different.

Printer	Resin	Printing Angle	Mean	St. Dev.	Min	Mdn	Max	Significance *
**Ray**	**Keyguide**	**0°**	17.574	1.33123	16.2312	17.1313	19.7551	A
**Ray**	**Keyguide**	**45°**	16.391	2.41588	13.2871	16.6735	19.5758	A
**Ray**	**Keyguide**	**90°**	17.0582	2.86991	12.7327	18.8119	19.3469	A
**Ray**	**C&B**	**0°**	24.7366	1.97465	22.5102	25.608	27.0303	B, C
**Ray**	**C&B**	**45°**	24.9361	1.22803	23.2245	24.8844	26.6931	B, C
**Ray**	**C&B**	**90°**	26.3785	5.16328	20.8812	27.2143	31.7525	B, C
**Night**	**Keyguide**	**0°**	28.3125	4.88117	24.4257	27.0891	36.5354	C, D
**Night**	**Keyguide**	**45°**	30.038	0.66664	29.2449	30.0297	30.8119	C, D
**Night**	**Keyguide**	**90°**	20.405	0.97769	19.0792	20.6432	21.5612	A, B
**Night**	**C&B**	**0°**	34.6666	3.55732	30.5446	34.7245	38.8911	C, D
**Night**	**C&B**	**45°**	27.4479	1.62139	24.7538	27.7857	28.8586	C
**Night**	**C&B**	**90°**	28.5209	3.94747	24.2323	28.5126	32.6436	C

**Table 5:** Descriptive statistics inherent to flexural strength. The unit of measurement of the results obtained is in megapascals (MPa). * means that values with the same letters are not significantly different.

Printer	Resin	Printing Angle	Mean	St. Dev.	Min	Mdn	Max	Significance *
**Ray**	**Keyguide**	**0°**	69.19766	5.24172	63.91017	67.45454	77.78571	A
**Ray**	**Keyguide**	**45°**	64.53951	9.51250	52.31806	65.65178	77.07954	A
**Ray**	**Keyguide**	**90°**	67.16659	11.30025	50.13490	74.07178	76.17857	A
**Ray**	**C&B**	**0°**	97.40052	7.77518	88.63392	100.83165	106.43181	B, C
**Ray**	**C&B**	**45°**	98.18579	4.83537	91.44642	97.98241	105.10396	B, C
**Ray**	**C&B**	**90°**	103.86527	20.33041	82.21967	107.15625	125.02537	B, C
**Night**	**Keyguide**	**0°**	111.48055	19.21961	96.17636	106.66336	143.85795	B, E
**Night**	**Keyguide**	**45°**	118.27452	2.62488	115.15178	118.24195	121.32178	B, E
**Night**	**Keyguide**	**90°**	80.34486	3.84963	75.12438	81.28266	84.89732	A, C
**Night**	**C&B**	**0°**	136.49971	14.00695	120.26918	136.72767	153.13366	D
**Night**	**C&B**	**45°**	108.07622	6.38420	97.46796	109.40625	113.63068	B, E
**Night**	**C&B**	**90°**	112.30100	15.54314	95.41477	112.26821	128.53403	B, D, E

**Table 6:** Descriptive statistics inherent to the modulus of elasticity (E). The unit of measurement of the results obtained is in megapascals (MPa). * means that values with the same letters are not significantly different.

Printer	Resin	Printing Angle	Mean	St. Dev.	Min	Mdn	Max	Significance *
**Ray**	**Keyguide**	**0°**	328.55559	89.53280	194.82730	351.40071	418.35329	A
**Ray**	**Keyguide**	**45°**	430.95389	268.01140	203.10394	295.30420	807.49981	A
**Ray**	**Keyguide**	**90°**	275.93538	83.12898	177.64188	276.93983	389.92806	A
**Ray**	**C&B**	**0°**	1484.31417	212.20628	1196.90684	1432.75721	1731.92789	B, C, F
**Ray**	**C&B**	**45°**	1494.97383	144.67238	1315.41197	1546.75568	1663.53232	B, C, D, F
**Ray**	**C&B**	**90°**	2026.84670	450.34765	1569.85383	1854.83836	2670.77600	D, E, G
**Night**	**Keyguide**	**0°**	1294.28775	173.72961	1107.53839	1276.56391	1579.30196	B, C
**Night**	**Keyguide**	**45°**	1321.44351	60.66754	1219.81604	1327.32230	1371.34584	B, C
**Night**	**Keyguide**	**90°**	988.34151	52.38467	929.47640	1019.19234	1035.04458	B
**Night**	**C&B**	**0°**	1678.67238	254.87093	1407.05243	1785.74370	1960.18841	C, F, G
**Night**	**C&B**	**45°**	2353.31490	308.62500	2008.83560	2462.9126	2741.56766	E
**Night**	**C&B**	**90°**	1879.03858	427.49913	1197.04412	1954.87031	2357.20909	F, E, G

**Table 7:** Linear regressions of the variables used in this study. *: *p* < 0.05.

Dependent Variable	Independent Variable	*p*-Value
Deflection at 0.1 mm	Material	0.544
	Degree	0.111
	Printer	0.0338 *
Deflection at 0.2 mm	Material	0.782
	Degree	0.064
	Printer	0.0206 *
Maximum Load	Material	*p* < 0.0001 *
	Degree	0.914
	Printer	0.000303 *

## Data Availability

The original contributions presented in the study are included in the article, further inquiries can be directed to the corresponding author.
